# Differences between Drug-Induced and Contrast Media-Induced Adverse Reactions Based on Spontaneously Reported Adverse Drug Reactions

**DOI:** 10.1371/journal.pone.0142418

**Published:** 2015-11-06

**Authors:** JiHyeon Ryu, HeeYoung Lee, JinUk Suh, MyungSuk Yang, WonKu Kang, EunYoung Kim

**Affiliations:** 1 Evidence-Based Research Laboratory, Department of Clinical Pharmacy and Pharmaceutical Care, College of Pharmacy, Chung-Ang University, Seoul, South Korea; 2 Department of Pharmacy, Division of Pharmacovigilance, Saint Mary’s Hospital, Daejeon, South Korea; 3 Department of Pharmaceutical Industry, ChungAng University, Seoul, South Korea; 4 College of Pharmacy, Chung-Ang University, Seoul, South Korea; Kinki University Faculty of Medicine, JAPAN

## Abstract

**Objective:**

We analyzed differences between spontaneously reported drug-induced (not including contrast media) and contrast media-induced adverse reactions.

**Methods:**

Adverse drug reactions reported by an in-hospital pharmacovigilance center (St. Mary’s teaching hospital, Daejeon, Korea) from 2010–2012 were classified as drug-induced or contrast media-induced. Clinical patterns, frequency, causality, severity, Schumock and Thornton’s preventability, and type A/B reactions were recorded. The trends among causality tools measuring drug and contrast-induced adverse reactions were analyzed.

**Results:**

Of 1,335 reports, 636 drug-induced and contrast media-induced adverse reactions were identified. The prevalence of spontaneously reported adverse drug reaction-related admissions revealed a suspected adverse drug reaction-reporting rate of 20.9/100,000 (inpatient, 0.021%) and 3.9/100,000 (outpatients, 0.004%). The most common adverse drug reaction-associated drug classes included nervous system agents and anti-infectives. Dermatological and gastrointestinal adverse drug reactions were most frequently and similarly reported between drug and contrast media-induced adverse reactions. Compared to contrast media-induced adverse reactions, drug-induced adverse reactions were milder, more likely to be preventable (9.8% vs. 1.1%, *p <* 0.001), and more likely to be type A reactions (73.5% vs. 18.8%, *p <* 0.001). Females were over-represented among drug-induced adverse reactions (68.1%, *p <* 0.001) but not among contrast media-induced adverse reactions (56.6%, *p* = 0.066). Causality patterns differed between the two adverse reaction classes. The World Health Organization–Uppsala Monitoring Centre causality evaluation and Naranjo algorithm results significantly differed from those of the Korean algorithm version II (*p <* 0.001).

**Conclusions:**

We found differences in sex, preventability, severity, and type A/B reactions between spontaneously reported drug and contrast media-induced adverse reactions. The World Health Organization–Uppsala Monitoring Centre and Naranjo algorithm causality evaluation afforded similar results.

## Introduction

According to the World Health Organization (WHO), an adverse drug reaction (ADR) is a noxious, unintended, and often unavoidable response to normal therapeutic doses of a medicine [1]. The hospital admission rate due to ADRs is over 10% in some countries, and is associated with marked socioeconomic loss [2,3]. Detecting and establishing preventive measures against ADRs is essential for patient safety. Therefore, the importance of pharmacovigilance (PV) must be emphasized. Furthermore, an automatic or spontaneous reporting system is necessary to uncover ADRs [1]. Several ADR reporting and monitoring systems, including computerized surveillance systems, have encouraged the monitoring of ADRs at in-hospital regional PV centers, and could promote the early identification or prevention of ADRs with properly designed ADR detection methods [4,5]. Periodical evaluation and analysis of reported ADRs filed during PV enhances the understanding of the ADR magnitude and patterns.

The critical role of contrast media (CM) in adverse reactions is evident from previous epidemiological studies [6]. CM may be categorized as drugs, although safety information is lacking [6], particularly in Korea. The information about CM-induced adverse reactions (CM-ADRs) through a spontaneous reporting system in hospitals helps define the safety of CM after marketing [6]. Various evaluation tools have been developed and used to analyze ADRs. However, these tools have not been adapted to CM-ADRs [6,7]. These evaluation tools would be helpful to understand CM-ADRs and to compare them with non-CM induced ADRs (D-ADRs) [7].

ADR causality is a key issue in ADR evaluations. There is no universally accepted gold standard for causality assessment, although several tools have been developed for this purpose. Thus, discrepancies may exist between these causality tools [4].

The purpose of this study was to evaluate ADR patterns using the spontaneous ADR reporting system of an in-hospital PV center, and to differentiate between D-ADRs and CM-ADRs. The differences between D-ADRs and CM-ADRs were investigated based on the overall ADR patterns, clinical patterns, frequency, causality, severity, and preventability. Additionally, we analyzed the trends of the causality results from the three tools used in this study for each D-ADRs and CM-ADRs.

## Materials and Methods

### Data collection and study design

This study was conducted at St. Mary’s Hospital, a 660-bed facility in Daejeon, Republic of Korea. The hospital is a branch of the regional PV center that recently developed a computerized ADR reporting system. Following implementation of the computerized system in June 2010, spontaneously reported ADRs were retrospectively collected by reviewing the electronic medical record (EMR) charts. The data were classified as either D-ADRs or CM-ADRs. D-ADRs were defined as adverse reactions induced by other drugs except CM. D-ADR data were collected from June 2010 (D-ADRs program launch) to August 2012, and CM-ADR data were collected from January 2011 (CM-ADRs program launch) to August 2012. Cases with insufficient data for evaluation, such as mistakenly reported data or data generated by system errors, were excluded.

The data collected included medication history, progress notes, medication orders, clinical consultation records, nursing records, and laboratory records. Additional data collected for each patient included sex, age, nature of the hospital visit (in- or outpatient), admission department, disease status, chief complaint, list of ailments, major signs and symptoms, medications, and clinical changes before and after drug administration.

Two clinical pharmacists with special training in ADRs evaluated the cases independently using objective criteria. In case of disagreement on the ADR categorization, a final determination was made after conferring with a third clinical pharmacist, and one allergic physician, who are the members on the ADR multidisciplinary team in-hospital PV center. The ADRs and the previous evaluations were subsequently stored in the hospital’s computer system. However, to ensure highest objectivity in the evaluation results, the two clinical pharmacists were blinded to each case’s assessment results, and received only the initial reports from a professional (nurse, doctor, pharmacist, or radiological technician) who suspected and reported the ADR. They evaluated each case independently.

### Ethics Statement

The Institutional Review Board (IRB) and the Ethics Deliberation Committee of St. Mary’s Hospital in Daejon approved this study protocol and all procedures conducted in this study. In the retrospective chart review, data and patient records were anonymized and de-identified prior to analysis and coded with an arbitrary number that was not linked to the subject. Written informed consents were exempted from the IRB. The research data were stored separately and were password protected.

### ADR evaluation

ADRs were defined according to WHO standards [1]. We evaluated the number of admissions related to ADRs (with or due to ADRs). To calculate a true prevalence of ADR-associated admissions, prospective screening of all patients for ADRs is required. Therefore, in this study setting, we calculate a prevalence of spontaneous reports of suspected ADRs associated admissions, instead. An “admission with a suspected ADR” referred to patients who visited the hospital for other diagnostic purposes and who had ADR-related hospitalizations after receiving medical treatment. An “admission due to a suspected ADR” referred to patients whose hospital visit was due to a suspected ADR. The causality, severity, preventability, and reaction types were analyzed for all ADRs. The ADR symptoms were coded using the Korea Food and Drug Administration (KFDA) WHO Adverse Reactions Terminology (WHO-ART) and were categorized based on the target organs using the Micromedex Healthcare Series adverse reaction categories [8,9]. The causative drugs were classified according to the Anatomical Therapeutic Chemical system and the Defined Daily Dose (ATC/DDD) category of 2012 [10].

Several decision aids for ADR causality grading have been published. Thus, there is no universally accepted gold standard for causality assessments. In this study, the confidence level of causality associated with the ADR agents was determined using the WHO-Uppsala Monitoring Centre (UMC) causality evaluation and the Naranjo algorithm (scale) [1,11–13], which are widely accepted tools in PV. In addition, the Korean algorithm version II was used. Each D-ADR and CM-ADR was analyzed and the trends within the causality results among the three tools were compared. Causal agents were categorized either as “possible” or as having a greater likelihood of causality (such as “probable” or “certain”), according to the data from this study.

The ADR severity evaluations were carried out using four commonly applied tools, i.e., KFDA severity [14], serious ADRs [15], the LDS scale [16], and the National Coordinating Council for Medication Error Reporting and Prevention (NCCMERP) [17]. Schumock and Thornton’s preventability criteria were used to evaluate the preventability of ADRs [18]. These criteria consist of seven questions, which evaluate the preventability or avoidance of ADRs, such as medication errors. The ADRs were further classified into type A or B reactions based on their underlying mechanisms [19]. The characteristics of the admission type or demographics for D-ADRs and CM-ADRs were compared to identify differences between the two types.

### Statistical analysis

Descriptive statistics were performed using Microsoft Office Excel 2010 (Microsoft Corporation, Redmond, WA, USA). Inferential statistical analyses were conducted using the Statistics for the Social Sciences Package (SPSS) software (version 19; IBM Corporation, Armonk, NY, USA). Pearson’s chi-squared test and Fisher’s exact test were used to analyze the differences in ADR causality and severity. Bonferroni correction was conducted to correct for multiple comparisons [20]. The kappa value was used to analyze the degree of agreement between the two reviewers, as well as between the hospital results and this study, based on the WHO-UMC causality evaluation of ADRs. Two-tailed tests were used *p <* 0.05 was considered significant.

## Results

In total, 1,335 cases of D-ADRs, CM-ADRs, and past drug allergies were reported during the study period. Following the implementation of the computerized system in June 2010, a total of 636 cases were recorded: 351 D-ADRs over 26 months and 285 CM-ADRs over 19 months. Twenty three reports with dubious causality were excluded from D-ADR cases. Among the CM-ADRs, 19 cases were excluded because two cases were system tests, and for the others, no patient visit record existed or no prescription of the suspected drugs in the EMR was due to the reporter’s error. In the final analysis, 328 D-ADRs and 266 CM-ADRs were included. The daily spontaneous reports increased over 3 years (0.8 cases/day, 2010; 1.6 cases/day, 2011; and 1.9 cases/day, 2012).

Females were over-represented among drug-induced adverse reactions (68.1%, *p <* 0.001) but not among contrast media-induced adverse reactions (56.6%, *p* = 0.066, Table 1). Seventy percent of D-ADRs occurred in in-patients, while only 21% of CM-ADRs were seen in in-patients (*p <* 0.001). The mean age did not differ between CM-ADRs and D-ADRs (*p* = 0.584, Table 1).

**Table 1:** Patient characteristics.

ADR Types		D-ADRs		CM-ADRs		D-ADRs vs.CM-ADRs
		Total	*p*-value^a^	Total	*p*-value^a^	*p*-value^a^
*Admission type*			<0.001		<0.001	<0.001
	Inpatients	231		57		
	Outpatients	97		209		
*Gender (%)*			<0.001		0.066	<0.001
	Male	104 (31.9)		118 (44.4)		
	Female	224 (68.1)		148 (55.6)		
*Age (year)*						0.584
	Mean ± SD	50.9 ± 19.7	-	51.4 ± 16.6	-	
	Range	0–94	-	3–83	-	-

ADR, adverse drug reaction; D-ADRs, drug-induced adverse drug reaction (not including contrast media adverse reaction); CM-ADRs, contrast media-induced adverse drug reactions.

^a^ Chi-square test

### D-ADRs

During the study period spontaneous reports of suspected D-ADRs associated hospital admissions were evaluated. The total prevalence of spontaneous reports of suspected D-ADRs related admissions were 20.3 and 3.9 cases per 100,000 admissions during the study period for inpatients and outpatients, respectively (Table 2).

**Table 2:** The prevalence of spontaneous reports of suspected D-ADRs related admissions during the study period.

Sites	Admission Types^a^	D-ADRs, n	Prevalence (cases per 100,000 admissions)
*In-patient department*			
	due to suspected D-ADRs	8	13.5
	with suspected D-ADRs	4	6.8
	Total	12	20.3
*Out-patient department*			
	due to suspected D-ADRs	21	1.9
	with suspected D-ADRs	22	2
	Total	43	3.9

D-ADRs: drug-induced adverse drug reactions (not including contrast media adverse drug reactions)

^a^ Admission with suspected ADR refers to patients who visited the hospital for other diagnostic purposes and had ADR-related hospitalizations after receiving medical treatment; admission due to suspected ADR refers to patients who visited the hospital because of ADRs.

A total of 109 drugs caused D-ADRs. Neurological drugs, such as tramadol, pethidine and fentanyl, showed the highest ADR frequency (40.9%), followed by antibiotics, including cephalosporin and vancomycin (27.1%) (Table 3). Based on the WHO-ART, 53 adverse drug event categories were identified as D-ADR symptoms. Dermatological reactions were the most common (35.2%), followed by gastrointestinal (33.2%) and neurological (14.6%) reactions. The number of ADR symptoms was greater than the total number because some reports included two or more symptoms (Table 4).

**Table 3:** The anatomical therapeutic chemical (ATC) classification of drugs involved in drug-induced adverse reactions (D-ADRs).

ATC	Medication (n)	n (%)
N	Tramadol (89), pethidine (24), fentanyl patch (5), tramadol (2), acetaminophen, alprazolam, amitriptyline, clonazepam, choline alfoscerate, diazepam, fentanyl injection, midazolam, oxcarbazepine, oxiracetam, pregabalin, quetiapine, rivastigmine patch, zolpidem	134 (40.9)
J	Flomoxef (12), ceftriaxone (9), ciprofloxacin (9), levofloxacin (7), cefixime (6), amoxicillin/clavulanate (6), levofloxacin (4), vancomycin (4), ampicillin/sulbactam (3), cefazolin (3), cefcapene (3), cefditoren (2), cefoperazone/sulbactam (2), cefoxitin (2), ceftizoxime (2), ethambutol (2), anti-tubercular agents, cefepime, cefotaxime, ceftezole, cefuroxime, clarithromycin, doxorubicin, doxycycline, isepamycin, isonicotinic acid, roxithromycin, sulfamethoxazole/trimethoprim	89 (27.1)
M	Allopurinol (4), ketorolac (4), aceclofenac (2), diclofenac (2), ibandronate (2), nimesulide (2), afloqualone, benzbromarone, celecoxib, eperison, mefenamic acid, risedronate, tizanidine, trypsin, zaltoprofen	25 (7.6)
R	Doxofylline (11), codeine (3), montelukast (3), formoterol (2), salmeterol/fluticasone (2), tiotropium (2), acetylcysteine, levocetirizine	25 (7.6)
A	Ranitidine (4), hyoscine-N-butylbromide (2), metoclopramide (2), dimenhydrinate, domperidone, famotidine, lansoprazole, mosapride, multivitamins, sulfasalazine, pancreatin/simethicone, polyethylene glycol, thioctic acid, Trestan^®^	19 (5.8)
H	Methimazole (4), levothyroxine, prednisolone, propylthiouracil	7 (2.1)
Others	Amino acids, cilostazol, gabexate, glycerin fructose, nutritional combinations, phytonadione, ticlopidine, bisoprolol, cilnidipine, molsidomine, nifedipine, rosuvastatin, cisplatin, docetaxel, infliximab, peginterferon, alfuzosin, raloxifene, ornidazole	29 (8.7)

^a^ ATC, anatomical therapeutic chemical; N, nervous system; J, systemic anti-infective agents; M, musculoskeletal system; R, respiratory system; A, digestive system; H, systemic hormonal preparations (excluding sex hormones and insulin).

**Table 4:** Classification of adverse drug reactions (ADRs) according to the affected organ or system.

Type of ADR	D-ADRs			CM-ADRs		
	(328 cases, 512 reactions*)			(266 cases, 316 reactions*)		
	Rank	No. (%)	ADR manifestations (n)	Rank	No. (%)	ADR manifestations (n)
Dermatologic	1	180 (35.2)	Rash (70), pruritus (46), urticarial (33), injection-site related (10), diaphoresis (7), facial edema (5), edema (4), eruption (3), flushing (2), acne (1), alopecia (1), skin exfoliation (1)	1	211 (66.8)	Urticaria (156), skin reaction-L^#^(144), pruritus (116), rash (20), skin reaction-G^#^ (2)
Gastro-intestinal	2	170 (33.2)	Nausea (87), vomiting(57), abdominal pain (8), diarrhea (7), indigestion (4), xerostomia (2), blood in stool (1), gastroesophageal reflux (1)	2	50 (18.8)	Vomiting (49), nausea (1),
Neurologic	3	75 (14.6)	Dizziness (44), headache (13), asthenia (6), dizziness (5), consciousness decreased (2), somnolence (2), insomnia (2), anxiety (1)	3	25 (7.9)	Dizziness (16), passed out (5),paralysis facial (4)
Respiratory	4	31 (6.1)	Dyspnea (29), cough (2)	5	9 (2.8)	Dyspnea (9)
Cardio-vascular	5	23 (4.5)	Hypotension (7), chest pain (4), palpitation (3), syncope (3), tachycardia (3,) chest discomfort (2), hypotension orthostatic (1)	4	11 (3.5)	Hypotension (9), hypertension (2)
Immuno- logic	6	7 (1.4)	Anaphylactoid reaction (6), anaphylactic shock (1)	6	4 (1.3)	Anaphylactic shock (4)
Others	others	26 (5.1)	Myalgia (6), neutropenia (5), abnormal LFT (5), Dysuria (3), hematuria (3), arthralgia (2), thrombocytopenia (1), hepatitis (1)	others	6 (1.9)	Fever (6)
Total		512* (100)			316* (100)	

^a^ ADRs, adverse drug reactions; D-ADRs, drug-adverse drug reactions (not including contrast media adverse drug reactions); CM-ADRs, contrast media-induced adverse drug reactions

*reactions reported, which maybe more than one for each reported case

^#^Skin reaction, L, localized; G, generalized

^**+**^Difference in rank.

**Fig 1:**
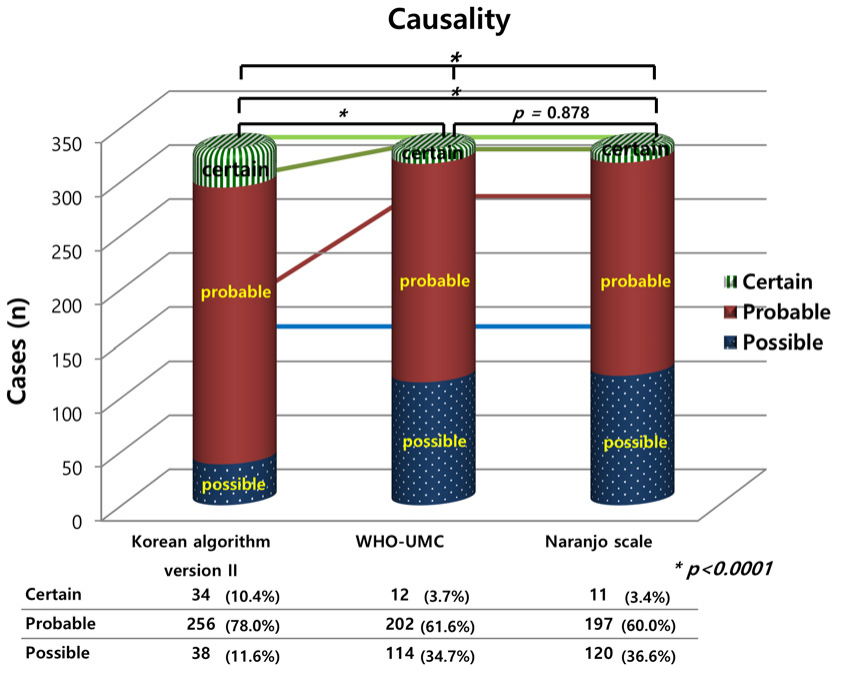
Characteristics of drug-induced adverse reactions (D-ADRs): causality versus evaluation tools. WHO-UMC, World Health Organization-Uppsala Monitoring Centre; D-ADRs, drug-induced adverse drug reactions (not including contrast media adverse drug reactions); * chi square test.

Causality assessment using the Korean Algorithm version II showed different trends compared to the WHO-UMC system and the Naranjo scale in D-ADRs (*p <* 0.001, Fig 1). The degree of conformity between our study and the previous hospital results was 0.937 as shown by the WHO- UMC causality evaluation of the D-ADRs.

The LDS scale mainly revealed mild events (79.3%). According to the NCC MERP criteria, 87.8% of the cases were category E (temporary harm requiring intervention). The preventable D-ADRs included 32 cases (9.8%) meeting the Schumock and Thornton’s preventability criteria, and the majority (241, 73.5%) of the D-ADRs were type A cases (Table 5). The degree of agreement between the two reviewers for the D-ADRs analysis was 0.917.

**Table 5:** Adverse drug reaction (ADR) characteristics.

Evaluation tool	Criteria		D-ADRs	CM-ADRs	D-ADRs vs. CM-ADRs*
			n = 328, n (%)	n = 266, n (%)	*p* value^b^
*Severity category*					
	Serious				<0.000
		Serious	25 (7.6)	46 (17.3)	
		Non-serious	303 (92.4)	220 (82.7)	
	Severity				<0.000
		Severe	9 (2.7)	46 (17.3)	
		Moderate	64 (19.5)	158 (59.4)	
		Mild	255 (77.7)	62 (23.3)	
	LDS scale				<0.000
		Severe	8 (2.4)	46 (17.3)	
		Moderate	60 (18.3)	157 (59.0)	
		Mild	260 (79.3)	63 (23.7)	
	NCC MERP				0.06
		Category E	288 (87.8)	219 (82.3)	
		Category F	40 (12.2)	47 (17.7)	
	Warning when re-prescribed				N/A
		Yes	113 (34.5)	N/A^a^	
		No	215 (65.5)		
*Preventability*					<0.000
	Preventable		32 (9.8)	3 (1.1)	
	Unpreventable		296 (90.2)	263 (98.9)	
*ADR type*					<0.000
	Type A		241 (73.5)	50 (18.8)	
	Type B		87 (26.5)	216 (81.2)	

ADR, adverse drug reaction; D-ADRs, drug-adverse drug reactions (not including contrast media adverse drug reactions); CM-ADRs, contrast media-induced adverse drug reactions

^a^ N/A, not available

^b^ Pearson’s chi-square test.

### CM-ADRs

**Fig 2:**
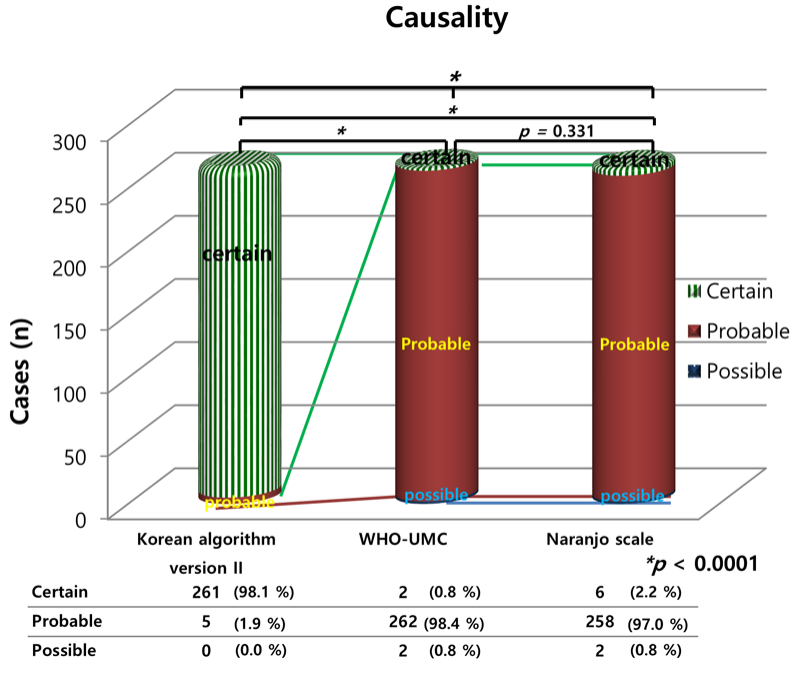
The characteristics of contrast media-induced adverse reactions: causality versus evaluation tool. WHO-UMC, World Health Organization-Uppsala Monitoring Centre; CM-ADRs, contrast media-induced adverse drug reactions;* chi square test.

The CM-ADR events included 240 cases (90.2%) with iopromide, 14 (5.4%) with iodixanol, 11 (4.1%) with gadobutrol, and one (0.4%) with iohexol. Dermatological reactions were the most common (66.8%) ADR, followed by gastrointestinal (18.8%) and neurological (7.9%) reactions (Table 4). Thirty-six patients were brought to the emergency room and their CM-ADRs represented 13.5% of all the reported CM-ADRs. The analysis of the spontaneous reports of admissions “due to suspected CM-ADRs” was possible only for the outpatient hospital visits in the current study setting because the patients required prompt adverse reaction treatment, which took place in the emergency department. For inpatients, CM are administered to them for diagnostic tests after admission due to some other chief complaint. In those cases, CM-ADR could occur and be captured. However, it was not CM-ADR-related admission. There were 79.9 spontaneous reports of suspected CM-ADR-related admissions due to CM-ADRs per 100,000 admissions. The results of the three causality tools are shown in Fig 2. The patterns of the causality results were similar between the WHO-UMC system and Naranjo scale, but differed from those obtained with the Korean algorithm (ver. II, *p <* 0.001).

The LDS scale analysis indicated that 76.3% of the CM-ADR cases were moderate to severe. The NCC MERP evaluation indicated that 82.3% (219) of them were category E events. Only three (1.1%) CM-ADRs were classified as preventable, and 216 events were classified as type B reactions. In 243 cases, treatment of adverse reactions required the use of drugs, including intravenous pheniramine, dexamethasone, normal saline hydration, furosemide, or oral hydroxyzine. Fluid intake and absolute rest were used as simple, supportive care for patients not requiring medication. The kappa value for the CM-ADR analysis was 1.0 between the two independent researchers.

### Comparisons between D-ADR and CM-ADR

The characteristic ADR patterns and patient demographics for D-ADRs and CM-ADRs were compared to determine any differences between the two types of ADRs reported. In the outpatients, the spontaneous reports of suspected D-ADR- and CM-ADR-related admissions were 3.9 and 79.9 per 100,000 admissions, respectively. The WHO-ART system revealed that the top three symptom patterns were similar between the D-ADRs and CM-ADRs (Table 4).

Dermatological reactions were the most common adverse events. Results from the three causality tools revealed that the WHO-UMC evaluation and Naranjo causality scale, but not the Korean algorithm (ver. II), were similar for both D-ADRs and CM-ADRs (Figs 1 and 2). All three causality tools indicated that the causal relationships were higher for the CM-ADRs than for the D-ADRs. The determination of the characteristics of the ADRs revealed significant differences in the severity evaluations between the D-ADRs and CM-ADRs except the NCC MERP results (Table 5). Most D-ADRs and CM-ADRs were non-preventable according to the Schumock and Thornton’s criteria, but this percentage was significantly higher for the CM-ADRs than for the D-ADRs (90.2% vs. 98.9%, *p <* 0.001). Similarly, the frequency of type B reactions was significantly higher for the CM-ADRs than for the D-ADRs (81.2% vs. 26.5%, *p <* 0.001).

## Discussion

We analyzed D-ADRs and CM-ADRs and evaluated their differences. Additionally, we compared the trends indicated by the ADR evaluation tools using spontaneously reported ADRs collected from a newly implemented computerized surveillance system at an in-hospital PV center. During the study period, 636 ADRs were reported over the course of 2 years. However, prior to the development of the computerized ADR program, only 28 spontaneous ADR reports were recorded over a three-year period. This improvement may primarily be attributed to national efforts, which include financial support from the KFDA [4, 7, 12]. Over the past three years, nine million spontaneous ADRs have been reported in Korea, ranking it fifth after Singapore, the United States of America, New Zealand, and Ireland [21]. Well-developed computer programs also facilitate and enhance ADR reporting. Our analysis of spontaneous reports of D-ADR related admissions revealed a reporting rate of suspected D-ADRs in inpatient and outpatient treatments in 20.9 (0.021%) and 3.9 (0.004%) cases per 100,000 hospital visits, respectively. These values are significantly lower than those reported in prospective observational studies, which ranged from about 0.16% to 15.7% for inpatient cases [22]. Admissions related to adverse reactions may not be clinically recognized, and those that are may not always be reported [23]. A true prevalence of ADR-related admissions cannot be calculated without the prospective screening of all patients for ADRs. Therefore, the results of our current spontaneous reporting study and those of prospective observational studies are not directly comparable.

On the other hand, this result may suggest that our newly implemented spontaneous reporting system still has a high level of under-reporting of ADRs despite the successful improvements over traditional paper-based reporting systems. Especially, CM-ADRs were less frequent in inpatients than D-ADRs (21.4% vs. 70.4%) in this study. This finding seems counter-intuitive, since contrast agents are usually given in the hospital setting. Though the reason for this observation is not clear, a possible explanation may be that there are several suspected causes of adverse events among inpatients who use contrast media, and the three most frequently reported CM-ADRs (dermatological, gastrointestinal, and neurological reactions) are similar to D-ADRs. Therefore, CM-ADRs may not be suspected, leading to under-reporting. Additionally, clinical information on inpatients is more detailed and reliable, and inpatients with significant risk factors could be better prepared to avoid adverse reactions before exposure to contrast media. To improve the reporting rate in the future, the promotion of and education on spontaneous ADR reports for our hospital members should be intensified and maintained. The concurrent computer-based ADR monitoring system using ADR detection signals also allows more efficient reporting [4].

The ADR data analysis in the current study included a number of factors, including the patients’ sex. The sex of an individual is considered a risk factor for developing ADRs. Several factors may explain these sex-related variances, including differences in pharmacodynamics or pharmacokinetics, hormonal levels, body weight, drug therapy compliance, compliance rate, and immunological factors [24–26]. The previously reported results were consistent with the ones obtained in the study, which showed that female sex was a risk factor for D-ADRs [24–26]. Indeed, the proportion of women with D-ADRs, was almost twice that of men (68.1% vs. 31.9%, *p <* 0.001). However, the CM-ADR evaluations revealed no significant differences between men and women (44.4% vs. 56.6%, *p* = 0.066). In previous studies, the higher frequency of ADRs in women might have been attributable to characteristics other than sex, and certain drugs did not show sex-related differences [25, 27]. Other data suggest that females may have a higher risk of type A ADRs [28]. In contrast, CM-ADRs are commonly considered type B ADRs, because they can occur even if a product is used appropriately [6]. Still controversy about the prevalence of type B reaction-related CM-ADRs according to sex exists. Although certain studies showed that females are at higher risk of developing CM-ADRs, others could not find significant differences between males and females [6, 29–31]. In this study, unavoidable type B ADRs were significantly higher in the CM-ADR than the D-ADR group (*p <* 0.001). In agreement with previous studies, the CM-ADRs were not significantly different between females and males in our study [6, 31].

Type B dermatologic reactions were reported most frequently in both groups and were 1.9 fold more prevalent for CM-ADRs than D-ADRs (66.8% vs 35.2%). In general, most CM-induced cutaneous reactions are allergic-like with immediate onset within one hour in comparison to those of D-ADR-induced reactions, which include late skin reactions [6, 31].

The ADR data from the current study were evaluated for causality using three assessment methods. In all three causality assessments, higher causality was identified in causal relationship between the CM-ADRs than between the D-ADRs. One of the reasons for these differences is that D-ADRs may include other suspected causes, i.e., D-ADRs may be caused or explained by the patients’ concurrent diseases, or other suspected drugs compared to those related to CM-ADRs. In this study, the results of the WHO-UMC evaluation and the Naranjo causality scale, but not those of the Korean algorithm (ver. II), were similar for the both D-ADRs and CM-ADRs. However, a previous study reported poor agreement between the Naranjo algorithm and WHO-UMC criteria [32]. In another study, “probable” or “certain” ADRs were scored more often when using the Korean algorithm (ver. II) than when using the Naranjo scale, which is similar to the findings of the current study [4]. Although the original version of the Korean algorithm has been revised to the current version II to improve its over-estimation bias, overestimation still persist to a certain degree [12]. Several studies have reported discrepancies between causality tools [4, 32–34]. Therefore, a future formal study on the differences between causality tools needs a separate design to address this issue. The WHO-UMC system was developed for international drug monitoring in consultation with the National Centers joining the program. The WHO-UMC is a definition-based practical assessment tool that considers the clinical pharmacological views and documented quality of the observation simultaneously [11]. The Naranjo algorithm [13] consists of a list of weighted questions, including those related to drug levels, previous adverse events with the medication, and the time-event relationship; this is particularly helpful for less experienced assessors, who may be dealing with unexpected or new medication-associated ADRs [13,35]. The Korean algorithm (ver. II) is a domestically developed tool, which has more detailed questions designed to clarify the causality [4,12]. Our current approach afforded us the advantages and disadvantages of the differences in each evaluation index, depending on the situation [36].

In this study, serious ADRs constituted 7.6% of the D-ADRs, similar to the 7.0% reported in a previous study [36]. However, the definition of serious adverse reactions differed between our and the previous study. Continued efforts to achieve consistency in these evaluations are necessary, and should include case education on the classification of serious ADRs. This education is essential since evaluator training and access to information may influence opinions. The KFDA adverse event severity, NCCMERP categories, and the LDS scale have different items and evaluation systems. It is, therefore, important to use a variety of metrics to account for any bias introduced by different evaluators. About 10% of the D-ADRs were preventable, which is lower than the 33% reported in previous studies [37, 38]. However, an objective comparison with other studies is difficult, because our study included spontaneously reported data.

CM-ADRs were more serious and severe than D-ADRs in this study, reflecting the use of CMs at much higher concentrations and doses than other intravascular drugs [6, 33]. However, preventable CM-ADRs were less reported than D-ADRs. A previous study has shown that specific preventive therapy reduced serious CM-ADRs and that CM-ADRs are far more promptly treated than ADRs caused by other drugs [6]. Therefore, a close cooperation between the radiology and clinical departments is required to reduce the number of CM-ADRs. Developing specific protocols for patients who require special treatments, which include sufficient liquid intake and premedication based on the medical history, may contribute to preventing CM-ADRs.

This study had several limitations. First, as mentioned above, it is difficult to determine the true prevalence of the adverse reactions because the data dependent on spontaneous reports. Moreover, extrapolation of these findings to other institutional situations may be difficult, because the study was based on spontaneous reports at a single institution. Additionally, our data analyses were conducted using EMR, rather than real-time analyses via direct patient interviews. For example, parameters such as diet, lifestyle, and other potential contributing factors that were not recorded in the existing ADR reports, were not evaluated in this study. In the future, more related and prospective studies may clarify and reduce these limitations.

Despite these limitations, we performed a comprehensive analysis of the ADRs reported at an in-hospital PV center during a three-year period. In particular, we performed separate analyses on the D-ADRs and CM-ADRs, whereas previous studies did not partition the data. This study also assessed the rate of the ADR-related admissions among cases of spontaneously reported ADRs. The D-ADRs and CM-ADRs were successfully evaluated using the newly implemented ADR program. The ADR-related admission rate from spontaneous reporting was lower than that found in previous studies. Accumulated data from properly developed ADR programs may facilitate future prospective studies aimed at preventing ADRs.
